# Occurrence, Dietary Exposure Scenarios and Risk Assessment of Aflatoxins from Dried Fruits and Chocolates in Armenia

**DOI:** 10.3390/foods15081329

**Published:** 2026-04-10

**Authors:** Davit Pipoyan, Meline Beglaryan, Yepraqsya Arshakyan, Bagrat Harutyunyan

**Affiliations:** Center for Ecological-Noosphere Studies, Abovyan 68, Yerevan 0025, Armenia; david.pipoyan@cens.am (D.P.); yepraqsya.arshakyan@cens.am (Y.A.); bagrat.harutyunyan@cens.am (B.H.)

**Keywords:** mycotoxins, food safety, regulatory gap, health concerns

## Abstract

This study aimed to estimate dietary exposure to aflatoxins (AFs) and characterize its associated risks through the consumption of dried fruits and chocolates among the adult population of Yerevan, the capital city of Armenia. Asflatoxin B1 (AFB1) and total AFs were determined using HPLC in 10 composite samples of widely consumed dried fruits and chocolates, prepared by pooling 100 individual sub-samples into 5 dried fruits and 5 chocolate composites. Individual consumption data were obtained via food frequency questionnaires and were stratified by consumer groups and percentiles. Exposure scenarios (lower-, middle-, upper-bound and detected mean) were applied, and risk was assessed using the margin of exposure (MOE) approach with a BMDL_10_ of 0.4 μg/kg bw/day. The study findings revealed that dried fruits had higher contamination levels (detected mean content of 10 μg/kg AFB1, 15 μg/kg total AFs) compared to chocolates (detected mean content of 0.5 μg/kg AFB1, and 0.9 μg/kg total AFs), resulting in lower MOE values despite smaller consumption quantities. Detectable AFs in dried fruits from open (street) markets exceeded the EU maximum limits, while Armenia currently lacks national regulatory limits for these products. MOEs were below 10,000 for most consumption groups, indicating a potential public health concern. This research emphasizes the urgent need for continuous monitoring and the establishment of harmonized national regulatory limits for AFs in dried fruits.

## 1. Introduction

Mycotoxin contamination remains a challenge for food safety and public health worldwide [1,2]. Although mycotoxins are a large and diverse group of toxic secondary metabolites, only a limited number have emerged as major public health concerns due to their frequent occurrence in food and potent toxic effects [3]. Among these, aflatoxins (AFs), produced mainly by *Aspergillus flavus* and *Aspergillus parasiticus*, are some of the most hazardous compounds [4,5]. The International Agency for Research on Cancer (IARC) has classified naturally occurring mixtures of AFs as carcinogenic to humans and the metabolite aflatoxin M1 (AFM1) in milk as possibly carcinogenic [6]. Aflatoxin B1 (AFB1), the most prevalent and potent in the AFs group, is classified as a Group 1 human carcinogen [7]. Due to its genotoxic and non-threshold carcinogenic properties, regulatory authorities have not established a tolerable daily intake, and international risk assessment frameworks emphasize that exposure should be reduced to the lowest level reasonably achievable to protect public health [8]. In this context, assessment of the health risks associated with aflatoxin contamination cannot rely solely on analytical measurements of hazard levels in food commodities, as consumption patterns critically influence exposure; hence, an integrated exposure-based approach is required [9]. This is particularly important for food categories for which regulatory limits are absent or inconsistent across different countries. Dietary exposure assessment, therefore, provides a scientifically robust framework for estimating population intake, identifying high-risk groups and supporting risk-based public health and food safety interventions.

Recent studies conducted in Armenia have reported the presence of AFs in several food categories, including cereals, nuts, spices and milk, with measurable dietary exposure among the population [10,11]. These findings indicate that aflatoxin contamination is a persistent food safety concern that may contribute to cumulative dietary exposure. Moreover, exposure estimates from these studies highlight risks among population subgroups characterized by higher consumption of specific commodities. Despite these findings, several potentially important dietary contributors remain insufficiently investigated. Dried fruits and chocolates represent two such critical food groups. These commodities are among the food products particularly vulnerable to aflatoxin contamination due to multiple factors throughout the food chain, including pre-harvest conditions, post-harvest handling, drying, storage and processing. Studies from global reviews have reported that the mentioned food items are commonly contaminated with aflatoxigenic species, primarily *Aspergillus flavus* and *Aspergillus parasiticus*, as well as other mycotoxigenic fungi, depending on environmental and storage conditions [12,13,14]. Moreover, dried fruits and chocolates are integral components of dietary habits and snacking practices [15,16]. Consequently, these commodities may substantially contribute to cumulative dietary exposure to aflatoxins, yet remain insufficiently considered in exposure-based risk assessment studies in Armenia. From a regulatory perspective, notable differences further highlight the need for such studies. In the European Union (EU), maximum levels have been established for AFB1 and for the sum of AFs (B1, B2, G1 and G2) in dried fruits [17]. In contrast, the Eurasian Economic Union (EAEU), of which Armenia is a member, currently only regulates AFB1 in certain confectionery products, including chocolate [18], and has not established limits for dried fruits or for total AFs in these product categories. In this regulatory context, exposure assessment is essential for estimating potential health risks and guiding consumer protection strategies tailored to national consumption habits and patterns.

Overall, despite the dietary relevance and susceptibility to aflatoxin contamination of dried fruits and chocolates, comprehensive investigations integrating contamination data with dietary exposure assessment in Armenia are currently lacking. Therefore, this study aimed to estimate dietary exposure to aflatoxins (AFs) and characterize the associated health risks through the consumption of dried fruits and chocolates among the adult population of Yerevan, the capital city of Armenia, where approximately one-third of the country’s population resides.

## 2. Materials and Methods

### 2.1. Sampling

Random sampling of dried fruits and chocolates was conducted in Yerevan, Armenia, following the principles of the total diet study (TDS) methodology [19,20], with the aim of obtaining representative composite samples reflecting the average dietary exposure of the adult population. Products were selected based on national food consumption data and market availability, prioritizing frequently consumed varieties commonly purchased by consumers. The sampling covered widely consumed dried fruits (major varieties including dried apricots, plums, figs, apples, and raisins) and chocolates (major varieties with different cocoa contents and ingredients). Sampling was carried out from October to November, 2023. A total of 100 individual sub-samples were randomly purchased from major retail markets and online grocery stores, ensuring the representation of leading brands and producers with substantial market presence. In addition, dried fruit samples were collected from open (street) markets, which are outside of official surveillance systems. Following the TDS methodology, individual sub-samples of each food category were pooled to generate 10 composite samples, including 5 dried fruit and 5 chocolate composites. Each composite sample was prepared by pooling at least 10 randomly selected individual sub-samples to ensure representative coverage of the product group. Since AFs can be heterogeneously distributed in food matrices, pooling multiple randomly collected individual sub-samples into composite samples was intended to reduce sampling variability and to provide an estimate of average contamination for the population. Although this approach may average out localized high contamination, it is more suitable for exposure assessment than for lot compliance evaluation.

### 2.2. AFs’ Quantification

Composite samples of dried fruits and chocolates were analyzed in the “Standard Dialog” laboratory, accredited under the ISO/IEC 17025 international standard for testing and calibration laboratories. The contents of AFB1 and total AFs (sum of B1, B2, G1 and G2) were determined using high-performance liquid chromatography (HPLC) methods following GOST standards [21,22]. Sample preparation, including clean-up, followed the procedures specified in these standards to ensure accurate determination of AFs in complex food matrices. Samples (10 g) were extracted with methanol–water (80:20, *v*/*v*), followed by filtration and clean-up using immunoaffinity columns specific for aflatoxins. The analyses were conducted using an HPLC system with a fluorescence detector. The chromatographic conditions were as follows: a C1 reverse-phase column (150 × 4.6 mm, 5 μm), a mobile phase consisting of water–methanol–acetonitrile (60:20:20, *v*/*v*/*v*), a flow rate of 1.0 mL/min, an injection volume of 20 μL and detection with post-column derivatization (excitation of 365 nm and emission of 435 nm). Certified reference standards of AFs (AFB1, AFB2, AFG1, AFG2) were obtained from a commercial supplier Sigma-Aldrich, USA. Standards were prepared in methanol (HPLC grade) and stored at −18 °C in the dark. Quality control procedures included analysis of blank samples, use of spiked recovery samples, and regular calibration verification. Measurement uncertainty was estimated according to EURACHEM guidelines, considering contributions from calibration, repeatability and recovery. The expanded uncertainty (k = 2) was found to be within ±15–25%, depending on the analyte and matrix. Recovery tests were performed using blank matrix samples spiked at three concentration levels for each aflatoxin. The spiking levels for AFB1 were 1.0, 5.0 and 10.0 µg/kg, for AFB2 were 0.5, 2.5 and 5.0 µg/kg, for AFG1 were 1.0, 5.0 and 10.0 µg/kg, and for AFG2 were 0.5, 2.5 and 5.0 µg/kg. These levels were selected to cover the regulatory limits and expected contamination range. The obtained recoveries ranged between 72% and 108%, depending on the matrix and analyte. The analytical method provided a limit of detection (LOD) of 0.1 μg/kg and a limit of quantification (LOQ) of 0.3 μg/kg.

### 2.3. Dietary Exposure and Risk Assessment

The deterministic risk assessment was conducted through the estimation of daily intake and margin of exposure (MOE) values for both AFB1 and total aflatoxins (AFs).

Dietary exposure to AFB1 and total AFs through the consumption of the studied food items (dried fruits, chocolates) was assessed by calculating the estimated daily intake (EDI):

(1) EDI = C_content_ × C_food_/BW

where EDI—estimated daily intake of AFs through consumption of the studied food item (μg/kg bw /day), C_content_—content of AFs in the food item (μg/kg), C_food_—daily consumption of the food item (kg/day), and BW—body weight (kg). In this study, the average body weight (BW) for the studied adult population was 70.48 kg [10].

EDI values were determined under several scenarios considering different approaches to left-censored data treatment using the LOQ value of 0.3 μg/kg [23]. Specifically, in the lower bound (LB) scenario, results below the limit of quantification (LOQ) were assigned a value of 0, in the middle bound (MB) scenario, results below the LOQ were replaced with a value equal to LOQ/2, and in the upper bound (UB) scenario, results below the LOQ were replaced with the LOQ value [23]. In addition, the detected mean (DM) was calculated using only samples with detectable and quantifiable levels.

EDI values were calculated using individual-based consumption data on dried fruits and chocolates derived from a previously conducted food frequency questionnaire (FFQ), an anonymous dietary survey among adult residents of Yerevan (*n* = 545, including 310 females and 235 males) aged 18–83 years [10]. For the present study, reported portion sizes and frequencies of dried fruit and chocolate consumption were used to estimate daily intake and associated exposure. To address different consumption patterns within the population, consumers of dried fruits and chocolates were classified into three groups using the Visual Binning tool in IBM SPSS Statistics (version 22.0). In addition to this consumer-group-based approach, percentile-based estimations were conducted including the 25th (P25), 50th (P50), 75th (P75) and 95th (P95) consumption percentiles, representing increasing levels of exposure from low to high consumers.

Risk characterization for AFB1 and total AF exposure through dried fruit and chocolate consumption was performed using the margin of exposure (MOE) approach [24]:

(2) MOE = RP/EDI

The benchmark dose lower confidence limit (BMDL_10_) of 0.4 μg/kg bw/day for AFB1 was used as the reference point (RP) in Equation (2) to calculate the MOE of AFs. This BMDL_10_, established by the EFSA, refers to the liver carcinogenicity posed by AFB1 exposure in rats [7]. MOE values were calculated for different exposure scenarios (LB, MB, UB, DM), consumption percentiles (P25, P50, P75, P95) and consumer groups (low, medium and high consumers). According to the EFSA risk assessment framework, and in view of the genotoxic and carcinogenic properties of aflatoxins, MOE values below 10,000 (MOE < 10,000) raise concerns for public health [7].

## 3. Results

### 3.1. Aflatoxins in Dried Fruits and Chocolates

**Figure 1:**
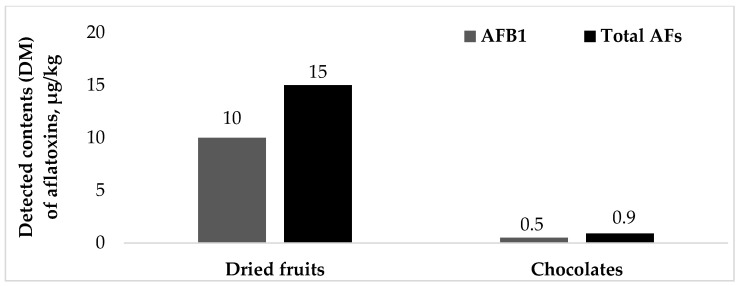
Mean detected contents (DM) of aflatoxin (AFB1 and total AFs) in the studied dried fruits and chocolates (μg/kg).

Aflatoxins were detected in one composite sample of dried fruits and in three composite samples of chocolate; however, the contamination levels were notably higher in dried fruits than in chocolate (Table 1). The LB, MB and UB substitution scenarios provide a realistic range of possible average contamination levels in the overall sample set by addressing the non-detected and non-quantified results. Figure 1 presents the mean detected contents (DM) of aflatoxin B1 (AFB1) and total aflatoxins (AFs) in the analyzed composite samples. The detected mean (DM) represents the mean calculated considering only positive samples with quantifiable aflatoxin levels, excluding non-detected and non-quantified (<LOQ) results. Because the detected mean for dried fruits was based on a single positive composite sample, calculation of a standard deviation (SD) was not possible for that product category. For chocolates, the AFB1 SEM was 0.1 μg/kg (0.5 ± 0.1 μg/kg), and for total AFs, it was 0.12 μg/kg (0.9 ± 0.12 μg/kg).

### 3.2. Estimated Daily Intake (EDI) and Margin of Exposure (MOE) of Aflatoxins

Dietary intake of AFB1 (Table 2 and Table 3) and total AFs (Table 4 and Table 5) through the consumption of dried fruits and chocolates was estimated using deterministic approaches, including LB, MB, UB and DM scenarios, to address the uncertainty associated with left-censored data (<LOQ). These approaches provide a comprehensive characterization of exposure, ranging from conservative to realistic and worst-case scenarios. The EDI values of AFB1 and total AFs varied depending on both contamination assumptions (i.e., LB, MB, UB and DM scenarios) and consumption levels.

Due to the absence of a safe threshold for genotoxic carcinogens, risk assessment of aflatoxins relies on the margin of exposure (MOE) approach, as recommended by the EFSA [7]. In the current study, risk characterization based on the MOE approach showed clear differences in aflatoxin-related health concerns associated with dried fruits and chocolates, reflecting consumption levels, consumer groups and percentiles (Table 6, Table 7, Table 8 and Table 9). MOE values below 10,000, derived from the ratio of the BMDL_10_ to estimated exposure, are considered to represent a possible concern for public health [7].

**Table 2:** Estimated daily intake (EDI) of AFB1 via consumption of dried fruits.

Studied Food Items	Consumer Groups	Consumption Levels (g/day)	Chronic EDI (μg/kg bw/day) of AFB1			
			*LB*	*MB*	*UB*	*DM*
Dried Fruits	All (*n* = 229 consumers)	11.88	3.37 × 10^−4^	3.57 × 10^−4^	3.78 × 10^−4^	1.69 × 10^−3^
	Group 1 (34% of consumers)	2.66 (*range* 1.23–4.24)	7.55 × 10^−5^ (3.49 × 10^−5^–1.20 × 10^−4^)	8.00 × 10^−5^ (3.70 × 10^−5^–1.28 × 10^−4^)	8.46 × 10^−5^ (3.91 × 10^−5^–1.35 × 10^−4^)	3.78 × 10^−4^ (1.75 × 10^−4^–6.02 × 10^−4^)
	Group 2 (33% of consumers)	7.65 (*range* 4.28–12.95)	2.17 × 10^−4^ (1.21 × 10^−4^–3.68 × 10^−4^)	2.30 × 10^−4^ (1.29 × 10^−4^–3.90 × 10^−4^)	2.43 × 10^−4^ (1.36 × 10^−4^–4.12 × 10^−4^)	1.09 × 10^−3^ (6.07 × 10^−4^–1.84 × 10^−3^)
	Group 3 (33% of consumers)	25.82 (*range* 13.37–50.4)	7.33 × 10^−4^ (3.79 × 10^−4^–1.43 × 10^−3^)	7.77 × 10^−4^ (4.02 × 10^−4^–1.52 × 10^−3^)	8.21 × 10^−4^ (4.25 × 10^−4^–1.60 × 10^−3^)	3.66 × 10^−3^ (1.90 × 10^−3^–7.15 × 10^−3^)
	P25	3.29	9.34 × 10^−5^	9.90 × 10^−5^	1.05 × 10^−4^	4.6710^-4^
	P50	7.25	2.06 × 10^−4^	2.18 × 10^−4^	2.30 × 10^−4^	1.03 × 10^−3^
	P75	16.04	4.55 × 10^−4^	4.83 × 10^−4^	5.10 × 10^−4^	2.28 × 10^−3^
	P95	40.41	1.15 × 10^−3^	1.22 × 10^−3^	1.28 × 10^−3^	5.73 × 10^−3^

Note: LB—lower bound, MB—middle bound, UB—upper bound, DM—detected mean, P25—25th percentile, P50—50th percentile, P75—75th percentile, P95—95th percentile.

**Table 3:** Estimated daily intake (EDI) of AFB1 via consumption of chocolates.

Studied Food Items	Consumer Groups	Consumption Levels (g/day)	Chronic EDI (μg/kg bw/day) of AFB1			
			*LB*	*MB*	*UB*	*DM*
Chocolates	All (*n* = 381 consumers)	33.25	1.42 × 10^−4^	1.70 × 10^−4^	1.98 × 10^−4^	2.36 × 10^−4^
	Group 1 (34% of consumers)	3.0 (*range* 0.8–5.62)	1.28 × 10^−5^(3.41 × 10^−6^–2.39 × 10^−5^)	1.54 × 10^−5^ (4.09 × 10^−6^–2.87 × 10^−5^)	1.79 × 10^−5^ (4.77 × 10^−6^–3.35 × 10^−5^)	2.13 × 10^−5^ (5.68 × 10^−6^–3.99 × 10^−5)^
	Group 2 (32% of consumers)	19.75 (*range* 5.92–44.72)	8.41 × 10^−5^(2.52 × 10^−5^–1.90 × 10^−4^)	1.01 × 10^−4^ (3.02 × 10^−5^–2.28 × 10^−4^	1.18 × 10^−4^ (3.53 × 10^−5^–2.67 × 10^−4^)	1.40 × 10^−4^(4.20 × 10^−5^–3.17 × 10^−4^)
	Group 3 (34% of consumers)	71.06 (*range* 44.99–149.95)	3.02 × 10^−4^(1.92 × 10^−4^–6.38 × 10^−4^)	3.63 × 10^−4^ (2.30 × 10^−4^–7.66 × 10^−4^)	4.23 × 10^−4^ (2.68 × 10^−4^–8.94 × 10^−4^)	5.04 × 10^−4^(3.19 × 10^−4^–1.06 × 10^−3^)
	P25	7.37	3.14 × 10^−5^	3.77 × 10^−5^	4.39 × 10^−5^	5.23 × 10^−5^
	P50	22.36	9.52 × 10^−5^	1.14 × 10^−4^	1.33 × 10^−4^	1.59 × 10^−4^
	P75	52.28	2.23 × 10^−4^	2.67 × 10^−4^	3.12 × 10^−4^	3.71 × 10^−4^
	P95	104.57	4.45 × 10^−4^	5.34 × 10^−4^	6.23 × 10^−4^	7.42 × 10^−4^

Note: LB—lower bound, MB—middle bound, UB—upper bound, DM—detected mean, P25—25th percentile, P50—50th percentile, P75—75th percentile, P95—95th percentile.

**Table 4:** Estimated daily intake (EDI) of total AFs via consumption of dried fruits.

Studied Food Items	Consumer Groups	Consumption Levels (g/day)	Chronic EDI (μg/kg bw/day) of Total AFs			
			LB	MB	UB	DM
Dried Fruits	All (*n* = 229 consumers)	11.88	5.06 × 10^−4^	5.26 × 10^−4^	5.46 × 10^−4^	2.53 × 10^−3^
	Group 1 (34% of consumers)	2.66 (*range* 1.23–4.24)	1.13 × 10^−4^ (5.24 × 10^−5^–1.80 × 10^−4^)	1.18 × 10^−4^(5.45 × 10^−5^–1.88 × 10^−4^)	1.22 × 10^−4^ (5.65 × 10^−5^–1.95 × 10^−4^)	5.66 × 10^−4^ (2.62 × 10^−4^–9.02^ × 10^−4^^)
	Group 2 (33% of consumers)	7.65 (*range* 4.28–12.95)	3.26 × 10^−4^ (1.82 × 10^−4^–5.51 × 10^−4^)	3.39 × 10^−4^(1.89 × 10^−4^–5.73 × 10^−4^)	3.52 × 10^−4^ (1.97 × 10^−4^–5.95 × 10^−4^)	1.63 × 10^−3^(9.11 × 10^−4^–2.76 × 10^−3^)
	Group 3 (33% of consumers)	25.82 (*range* 13.37–50.4)	1.10 × 10^−3^ (5.69 × 10^−4^–2.15 × 10^−3^)	1.14 × 10^−3^ (5.92 × 10^−4^–2.23 × 10^−3^)	1.19 × 10^−3^(6.15 × 10^−4^–2.32 × 10^−4^)	5.50 × 10^−3^ (2.85 × 10^−3^–1.07 × 10^−2^)
	P25	3.29	1.40 × 10^−4^	1.46 × 10^−4^	1.51 × 10^−4^	7.00 × 10^−4^
	P50	7.25	3.09 × 10^−4^	3.21 × 10^−4^	3.33 × 10^−4^	1.54 × 10^−3^
	P75	16.04	6.83 × 10^−4^	7.10 × 10^−4^	7.37 × 10^−4^	3.41 × 10^−3^
	P95	40.41	1.72 × 10^−3^	1.79 × 10^−3^	1.86 × 10^−3^	8.60 × 10^−3^

Note: LB—lower bound, MB—middle bound, UB—upper bound, DM—detected mean, P25—25th percentile, P50—50th percentile, P75—75th percentile, P95—95th percentile.

**Table 5:** Estimated daily intake (EDI) of total AFs via consumption of chocolates.

Studied Food Items	Consumer Groups	Consumption levels (g/day)	Chronic EDI (μg/kg bw/day) of Total AFs			
			LB	MB	UB	DM
Chocolates	All (*n* = 381 consumers)	33.25	2.64 × 10^−4^	2.93 × 10^−4^	3.21 × 10^−4^	4.40 × 10^−4^
	Group 1 (34% of consumers)	3.0 (*range* 0.8–5.62)	2.39 × 10^−5^ (6.36×10-^6^–4.47 × 10^−5^)	2.65 × 10^−5^(7.04 × 10^−6^–4.94 × 10^−5^)	2.90 × 10^−5^ (7.72 × 10^−6^–5.42 × 10^−5^)	3.98 × 10^−5^ (1.06 × 10^−5^–7.44 × 10^−5^)
	Group 2 (32% of consumers)	19.75 (*range* 5.92–44.72)	1.57 × 10^−4^ (4.70 × 10^−5^–3.55 × 10^−4^)	1.74 × 10^−4^(5.21 × 10^−5^–3.93 × 10^−4^)	1.91 × 10^−4^ (5.71 × 10^−5^–4.31 × 10^−4^)	2.62 × 10^−4^ (7.84 × 10^−5^–5.92 × 10^−4^)
	Group 3 (34% of consumers)	71.06 (*range* 44.99–149.95)	5.65 × 10^−4^ (3.57 × 10^−4^–1.19 × 10^−3^)	6.25 × 10^−4^ (3.96 × 10^−4^–1.32 × 10^−3^)	6.86 × 10^−4^(4.34 × 10^−4^–1.45 × 10^−3^)	9.41 × 10^−4^ (5.96 × 10^−4^–1.99 × 10^−3^)
	P25	7.37	5.86 × 10^−5^	6.49 × 10^−5^	7.12 × 10^−5^	9.77 × 10^−5^
	P50	22.36	1.78 × 10^−4^	1.97 × 10^−4^	2.16 × 10^−4^	2.96 × 10^−4^
	P75	52.28	4.15 × 10^−4^	4.60 × 10^−4^	5.04 × 10^−4^	6.92 × 10^−4^
	P95	104.57	8.31 × 10^−4^	9.20 × 10^−4^	1.01 × 10^−3^	1.38 × 10^−3^

Note: LB—lower bound, MB—middle bound, UB—upper bound, DM—detected mean, P25—25th percentile, P50—50th percentile, P75—75th percentile, P95—95th percentile.

**Table 6:** Margin of exposure (MOE) of AFB1 via consumption of dried fruits.

Studied Food Items	Consumer Groups	Consumption Levels (g/day)	MOE of AFB1			
			LB	MB	UB	DM
Dried Fruits	All (*n* = 229 consumers)	11.88	1186	1119	1059	237
	Group 1 (34% of consumers)	2.66 (*range* 1.23–4.24)	5297 (3324–11,459)	4997 (3136–10,811)	4730 (2968–10,232)	1059 (665–2292)
	Group 2 (33% of consumers)	7.65 (*range* 4.28–12.95)	1842 (1088–3293)	1738 (1027–3107)	1645 (972–2940)	368 (218–659)
	Group 3 (33% of consumers)	25.82 (*range* 13.37–50.4)	546 (280–1054)	515 (264–995)	487 (250–941)	109 (56–211)
	P25	3.29	4284	4042	3825	857
	P50	7.25	1944	1834	1736	389
	P75	16.04	879	829	785	176
	P95	40.41	349	329	311	70

Note: LB—lower bound, MB—middle bound, UB—upper bound, DM—detected mean, P25—25th percentile, P50—50th percentile, P75—75th percentile, P95—95th percentile.

**Table 7:** Margin of exposure (MOE) of AFB1 via consumption of chocolates.

Studied Food Items	Consumer Groups	Consumption Levels (g/day)	MOE of AFB1			
			LB	MB	UB	DM
Chocolates	All (*n* = 381 consumers)	33.25	2826	2355	2019	1696
	Group 1 (34% of consumers)	3.0 *(range* 0.8–5.62)	31,240 (16,720–117,459)	26,033 (13,933–97,882)	22,314 (11,943–83,899)	18,744 (10,032–70,475)
	Group 2 (32% of consumers)	19.75 (*range* 5.92–44.72)	4758 (2101–15,873)	3965 (1751–13,337)	3399 (1501–11,338)	2855 (1261–9524)
	Group 3 (34% of consumers)	71.06 (*range* 44.99–149.95)	1322 (627–2089)	1102 (522–1741)	945 (448–1492)	793 (376–1253)
	P25	7.37	12,743	10,619	9102	7646
	P50	22.36	4203	3502	3002	2522
	P75	52.28	1797	1498	1284	1078
	P95	104.57	899	749	642	539

Note: LB—lower bound, MB—middle bound, UB—upper bound, DM—detected mean, P25—25th percentile, P50—50th percentile, P75—75th percentile, P95—95th percentile.

**Table 8:** Margin of exposure (MOE) of total AFs via consumption of dried fruits.

Studied Food Items	Consumer Groups	Consumption Levels (g/day)	MOE of Total AFs			
			LB	MB	UB	DM
Dried Fruits	All (*n* = 229 consumers)	11.88	791	760	732	158
	Group 1 (34% of consumers)	2.66 (*range* 1.23–4.24)	3532 (2216–7640)	3396 (2131–7346)	3270 (2052–7074)	706 (443–1528)
	Group 2 (33% of consumers)	7.65 (*range* 4.28–12.95)	1228 (726–2195)	1181 (698–2111)	1137 (672–2033)	246 (145–439)
	Group 3 (33% of consumers)	25.82 (*range* 13.37–50.4)	364 (186–703)	350 (179–676)	337 (173–651)	73 (37–141)
	P25	3.29	2856	2746	2645	571
	P50	7.25	1296	1246	1200	259
	P75	16.04	586	563	542	117
	P95	40.41	233	224	215	47

Note: LB—lower bound, MB—middle bound, UB—upper bound, DM—detected mean, P25—25th percentile, P50—50th percentile, P75—75th percentile, P95—95th percentile.

**Table 9:** Margin of exposure (MOE) of total AFs via consumption of chocolates.

Studied Food Items	Consumer Groups	Consumption Levels (g/day)	MOE of Total AFs			
			LB	MB	UB	DM
Chocolates	All (*n* = 381 consumers)	33.25	1514	1367	1247	908
	Group 1 (34% of consumers)	3.0 (*range* 0.8–5.62)	16,736 (8957–62,924)	15,116 (8090–56,835)	13,782 (7377–51,820)	10,041 (5374–37,755)
	Group 2 (32% of consumers)	19.75 (*range* 5.92–44.72)	2549 (1126–8503)	2302 (1017–7680)	2099 (927–7003)	1529 (675–5102)
	Group 3 (34% of consumers)	71.06 (*range* 44.99–149.95)	708 (336–1119)	640 (303–1011)	583 (276–921)	425 (201–671)
	P25	7.37	6827	6166	5622	4096
	P50	22.36	2252	2034	1854	1351
	P75	52.28	963	870	793	578
	P95	104.57	481	435	396	289

Note: LB—lower bound, MB—middle bound, UB—upper bound, DM—detected mean, P25—25th percentile, P50—50th percentile, P75—75th percentile, P95—95th percentile.

## 4. Discussion

### 4.1. Occurrence of Aflatoxins (AFs)

In dried fruits, only the composite sample purchased from markets, including open (street) markets, contained detectable AF levels (10 μg/kg AFB1 and 15 μg/kg total AFs). These products were typically sold without hermetic packaging, labeling or clear traceability information regarding origin, producer and storage conditions. Open (street) markets often operate outside systematic official surveillance and routine food safety monitoring programs. Consequently, such products do not undergo regular laboratory testing or safety control. Overall, these findings are consistent with international studies, which show that dried fruits are highly susceptible to mycotoxin contamination. A global review [13] indicated that AFs are one of the widespread contaminants in dried fruits, such as apricots, figs, raisins, prunes and dates, across different climatic regions, with contamination influenced by pre-harvest fungal infection, inadequate drying and improper storage under high temperature and humidity. These conditions are also consistent with the ecophysiology of toxigenic *Aspergillus species*, particularly *A. flavus* and *A. parasiticus*, which can grow and produce AFs under warm and humid conditions, especially when drying is delayed or storage moisture is insufficiently controlled [13,25]. Country-specific investigations support this pattern, reporting comparable or even higher contamination levels. For example, studies in neighboring countries such as Turkey [26] and Iran [27], as well as in Morocco [28], Pakistan [29] and China [30], have reported measurable AF levels in dried fruits, often with frequent co-occurrence of other mycotoxins.

For chocolates, detectable AF levels were found in three composite samples, including products sold by weight and those containing dried fruits or nuts. The presence of AFs in these chocolates is likely due to contamination of raw ingredients, as nuts and dried fruits are particularly vulnerable to AFs [10,13]. Although chocolate production involves thermal processing, AFs are heat-stable and may persist in the final product [12,31]. The results of this study are consistent with international findings, showing that chocolates with higher cocoa content or additional ingredients such as nuts and dried fruits tend to have higher AF levels than plain milk or white chocolates [32,33,34].

From a regulatory perspective, among the two studied food categories, maximum levels (MLs) in the EU are established only for dried fruits. According to Commission Regulation (EU) 2023/915, MLs are set at 5 μg/kg for AFB1 and 10 μg/kg for total AFs (sum of B1, B2, G1 and G2) in dried fruits placed on the market for final consumers [17]. In the present study, the detected mean values in dried fruits (Figure 1) exceeded these regulatory MLs, with AFB1 (10 μg/kg) being twice as high and total AFs (15 μg/kg) 1.5 times higher than the established levels. In contrast, the overall LB, MB and UB mean contents remained below the MLs due to the predominance of non-detected (<LOD) samples. It is noteworthy that the EAEU technical regulation on food safety, applicable in Armenia as a member state of the EAEU, does not establish regulatory levels for either AFB1 or total AFs in dried fruits. However, it sets an allowable level of 5 μg/kg for AFB1 in chocolates [18]. The analyzed chocolate composite samples contained, on average, 0.5 μg/kg of AFB1 (Figure 1), well below the allowable level (5 μg/kg). Nevertheless, the presence of detectable AFs in these food items, even below the regulatory levels, may cumulatively contribute to dietary exposure and potential chronic health risks.

### 4.2. Dietary Intake and Risk Assessment of Aflatoxins (AFs)

While chocolates are generally consumed at higher quantities than dried fruits, the exposure estimates were consistently higher for dried fruits under all scenarios. This reflects the substantially higher levels of AFB1 and total AFs detected in composite samples of dried fruits, highlighting that both consumption and contamination data are critical for accurate dietary exposure assessment. Stratification by consumer groups and consumption percentiles revealed clear trends. Exposure increased with higher consumption, as expected, with individuals at the 95th percentile or in the high-consumption group (Group 3) having the highest AF intakes. For example, P95 consumers of dried fruits had AFB1 EDI values up to 5.73 × 10^−3^ μg/kg bw/day (DM scenario), whereas P95 chocolate consumers reached only 7.42 × 10^−4^ μg/kg bw/day. Similarly, high-consumption dried fruit consumers (Group 3) had more than three times higher EDI values than high-consumption chocolate consumers. Among the different exposure scenarios, the DM approach showed the highest exposure estimates, followed by the UB and MB scenarios, while the LB approach resulted in the lowest exposure estimates. This observation can be explained by the fact that the DM scenario considers only detected levels, which were relatively high in dried fruits, whereas the UB approach assigns an LOD value to non-detected samples.

The AFB1 and total AFs risk assessment results showed that for dried fruits, the MOE values were consistently far below the reference threshold of 10,000 across all consumption levels, consumer groups and percentiles, indicating a potential health concern. At the studied population level, the average consumption of dried fruits (approximately 11.9 g/day) resulted in MOE values for total AFs ranging from 158 to 791 under DM and LB exposure assumptions, respectively. These values are substantially below 10,000, indicating an insufficient safety margin at typical intake levels of dried fruits. MOE values above 10,000 were obtained only for the lowest consumption of dried fruits (Group 1, minimum intake of approximately 1.23 g/day) under the LB, MB and UB exposure scenarios. At this low intake level, the estimated exposure was sufficiently low to maintain an adequate safety margin. Similarly, for chocolates, MOE values were below the threshold of 10,000 for most consumption levels, indicating a potential health concern at typical and high intake levels. The average consumption of chocolates (approximately 33.25 g/day) resulted in MOEs for total AFs ranging from 908 to 1514 in the case of the DM and LB scenarios, respectively. MOE values above 10,000 were estimated only for Group 1 consumers at the lowest (0.8 g/day) and mean (3 g/day) consumption levels combined with all contamination assumptions (i.e., LB, MB, UB and DM scenarios).

## 5. Conclusions

This study presents the first integrated TDS-based assessment in Armenia combining analytical determination of AFs with consumption-based dietary exposure and MOE risk characterization for dried fruits and chocolates. The findings indicated that exposure levels varied considerably depending on consumption patterns and contamination assumptions (i.e., LB, MB, UB and DM scenarios). The estimated MOE values were below the threshold of 10,000 in almost all evaluated scenarios. Values exceeding 10,000 were obtained only in a single case corresponding to low consumers. For average and high consumers, MOE values consistently remained below 10,000, indicating a potential health concern. Between the two investigated products, dried fruits represented the most critical contributor to aflatoxin exposure. Despite being consumed in smaller quantities compared with chocolates, their higher contamination levels resulted in lower MOE values and consequently greater concern. These findings confirm that foods consumed in smaller amounts may still pose a greater exposure risk when contamination levels are notably high.

From a regulatory perspective, the study reveals an important gap. While chocolates are regulated for AFB1 in EAEU countries, including Armenia, dried fruits are not currently subject to specific MLs under the applicable framework. The detection of AFB1 and total AFs in dried fruits and associated risk assessment results highlighting a public health concern, therefore, provide strong scientific justification for expanding national regulatory limits to include both AFB1 and total AFs in dried fruits, harmonizing standards and strengthening surveillance systems.

Overall, the findings highlight the need for continuous and large-scale monitoring programs covering both retail chains and markets, improved storage and traceability practices, reinforced food safety management at the producer level, and implementation of preventive contamination control measures. In this context, this study provides a comprehensive evidence base from food safety and consumer protection perspectives and supports targeted regulatory and risk management actions aimed at mitigating aflatoxin exposure and protecting public health.

## Figures and Tables

**Table 1:** AFB1 and total AF contents (μg/kg) in the studied food items.

Studied Foods	Composite Samples (Origin/Type)	AFB1 (μg/kg)			Total AFs (μg/kg)		
		LB	MB	UB	LB	MB	UB
Dried fruits	Packaged dried fruits from supermarkets	<LOQ	LOQ/2	LOQ	<LOQ	LOQ/2	LOQ
	Dried fruits sold by the weight/kg in supermarkets	<LOQ	LOQ/2	LOQ	<LOQ	LOQ/2	LOQ
	Packaged dried fruits from minimarkets	<LOQ	LOQ/2	LOQ	<LOQ	LOQ/2	LOQ
	Dried fruits from online markets/grocery stores	<LOQ	LOQ/2	LOQ	<LOQ	LOQ/2	LOQ
	Unpackaged dried fruits from markets, including open (street) markets	10	10	10	15	15	15
	**Mean contents (μg/kg)**	**2.0**	**2.12**	**2.24**	**3.0**	**3.12**	**3.24**
Chocolates	Milk chocolates and chocolate bars	<LOQ	LOQ/2	LOQ	<LOQ	LOQ/2	LOQ
	Dark chocolates	<LOQ	LOQ/2	LOQ	<LOQ	LOQ/2	LOQ
	Chocolates sold by the weight/kg	0.4	0.4	0.4	0.8	0.8	0.8
	Chocolates with dried fruits and raisins	0.5	0.5	0.5	1.0	1.0	1.0
	Chocolates with nuts	0.6	0.6	0.6	1.0	1.0	1.0
	**Mean contents (μg/kg)**	**0.3**	**0.36**	**0.42**	**0.56**	**0.62**	**0.68**

Note: LOQ—limit of quantification, LB—lower bound (results below the LOQ were assigned a value of 0), MB—middle bound (results below the LOQ were replaced with a value equal to LOQ/2), UB—upper bound (results below the LOQ were replaced with the LOQ value).

## Data Availability

The original contributions presented in the study are included in the article, further inquiries can be directed to the corresponding author.

## Abbreviations

The following abbreviations are used in this manuscript:

AFB1	Aflatoxin B1
AFs	Aflatoxins
BMDL	Benchmark Dose Lower Confidence Limit
BW	Body Weight
DM	Detected Mean
EAEU	Eurasian Economic Union
EDI	Estimated Daily Intake
EFSA	European Food Safety Authority
EU	European Union
GOST	State Union Standard
IARC	International Agency for Research on Cancer
HPLC	High-Performance Liquid Chromatography
FFQ	Food Frequency Questionnaire
ISO/IEC	International Electrotechnical Commission/International Organization for Standardization
LB	Lower Bound
LOD	Limit of Detection
LOQ MB	Limit of Quantification Middle Bound
ML	Maximum Limit
MOE	Margin of Exposure
P25	25th percentile
P50	50th percentile
P75	75th percentile
P95	95th percentile
RP SD	Reference Point Standard Deviation
TDS	Total Diet Study
UB	Upper Bound
